# Case Report: Acute Fulminant Myocarditis and Cardiogenic Shock After Messenger RNA Coronavirus Disease 2019 Vaccination Requiring Extracorporeal Cardiopulmonary Resuscitation

**DOI:** 10.3389/fcvm.2021.758996

**Published:** 2021-10-29

**Authors:** Yongwhan Lim, Min Chul Kim, Kye Hun Kim, In-Seok Jeong, Yong Soo Cho, Yoo Duk Choi, Jong Eun Lee

**Affiliations:** ^1^Department of Cardiovascular Medicine, Chonnam National University Medical School/Hospital, Gwangju, South Korea; ^2^Department of Cardiothoracic Surgery, Chonnam National University Medical School/Hospital, Gwangju, South Korea; ^3^Department of Emergent Medicine, Chonnam National University Hospital, Gwangju, South Korea; ^4^Department of Pathology, Chonnam National University Medical School, Gwangju, South Korea; ^5^Department of Radiology, Chonnam National University Hospital, Gwangju, South Korea

**Keywords:** fulminant myocarditis, COVID-19, vaccination, extracorporeal membrane oxygenation, case report

## Abstract

Recently, myocarditis following messenger RNA (mRNA) coronavirus disease 2019 (COVID-19) vaccination has become an important social issue worldwide. According to the reports so far, myocarditis related to mRNA COVID-19 vaccination is rare and usually associated with a benign clinical course without intensive care or any sequelae of fulminant myocarditis. Here, we report a case of acute fulminant myocarditis and cardiogenic shock after the mRNA COVID-19 vaccination, requiring extracorporeal cardiopulmonary resuscitation. Clinicians should keep in mind the possibility of progression to fulminant myocarditis in patients who presented with suggestive symptoms or signs of myocarditis after the COVID-19 vaccination.

## Introduction

Coronavirus disease 2019 (COVID-19) vaccinations have been regarded as the most important solution to terminate the current pandemic, but various side effects after vaccination are also emerging as an issue that cannot be overlooked. Although the side effects after COVID-19 vaccination are mostly benign reactions, including pain at the injection site, fever, or myalgia (1), several serious adverse events including thrombosis with thrombocytopenia syndrome, Guillain Barre syndrome, myocarditis, and pericarditis have been also reported (2). Among these complications, myocarditis caused by the inactivated vaccine has also been reported in other vaccines, such as smallpox (3) or influenza (4). A previous study about the incidence of cardiovascular adverse events after smallpox and influenza vaccinations revealed that the rates of clinical or subclinical myocarditis and pericarditis after smallpox vaccinations are very low (~1:5,500) (5). According to the recent report of the Center for Disease Control (CDC), most patients with myocarditis that occurs after messenger RNA (mRNA) COVID-19 vaccination usually recover without sequelae, and thus, CDC continues to recommend COVID-19 vaccination for adolescents and young adults (1).

In this study, we describe a case of acute fulminant myocarditis presenting with cardiac arrest following the mRNA COVID-19 vaccination, rescued by extracorporeal cardiopulmonary resuscitation (ECPR). As opposed to the known usual benign course of mRNA COVID-19 vaccination-related myocarditis, the patient presented with very rapid progression to cardiac arrest. Therefore, clinicians should keep in mind the possibility of progression to fulminant myocarditis in patients who presented with suggestive symptoms or signs of myocarditis after the COVID-19 vaccination.

## Case Description

**Figure 1:**
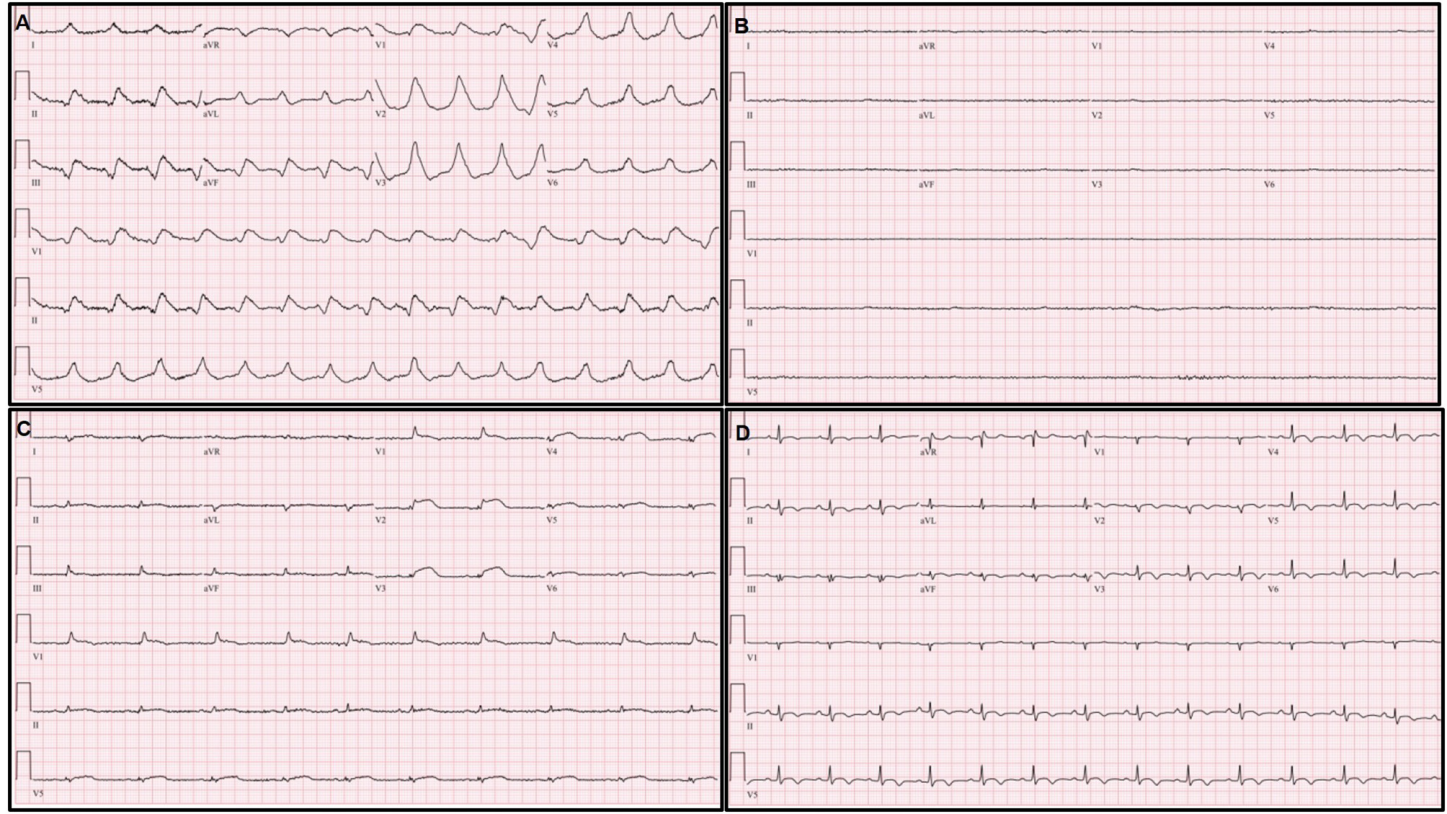
Serial changes of electrocardiography (ECG). **(A)** Extensive ST elevation with bizarrely wide QRS complexes immediately after extracorporeal membrane oxygenation (ECMO) application. **(B)** Ventricular and atrial electrical standstill during ECMO management. **(C)** Decreased but remained extensive ST elevation with narrowing and low voltage of QRS complexes on ECG on the third hospital day. **(D)** Normalization of ST segment elevation and QRS width; an increased but remained low voltage QRS complex on pre-discharge ECG.

**Figure 2:**
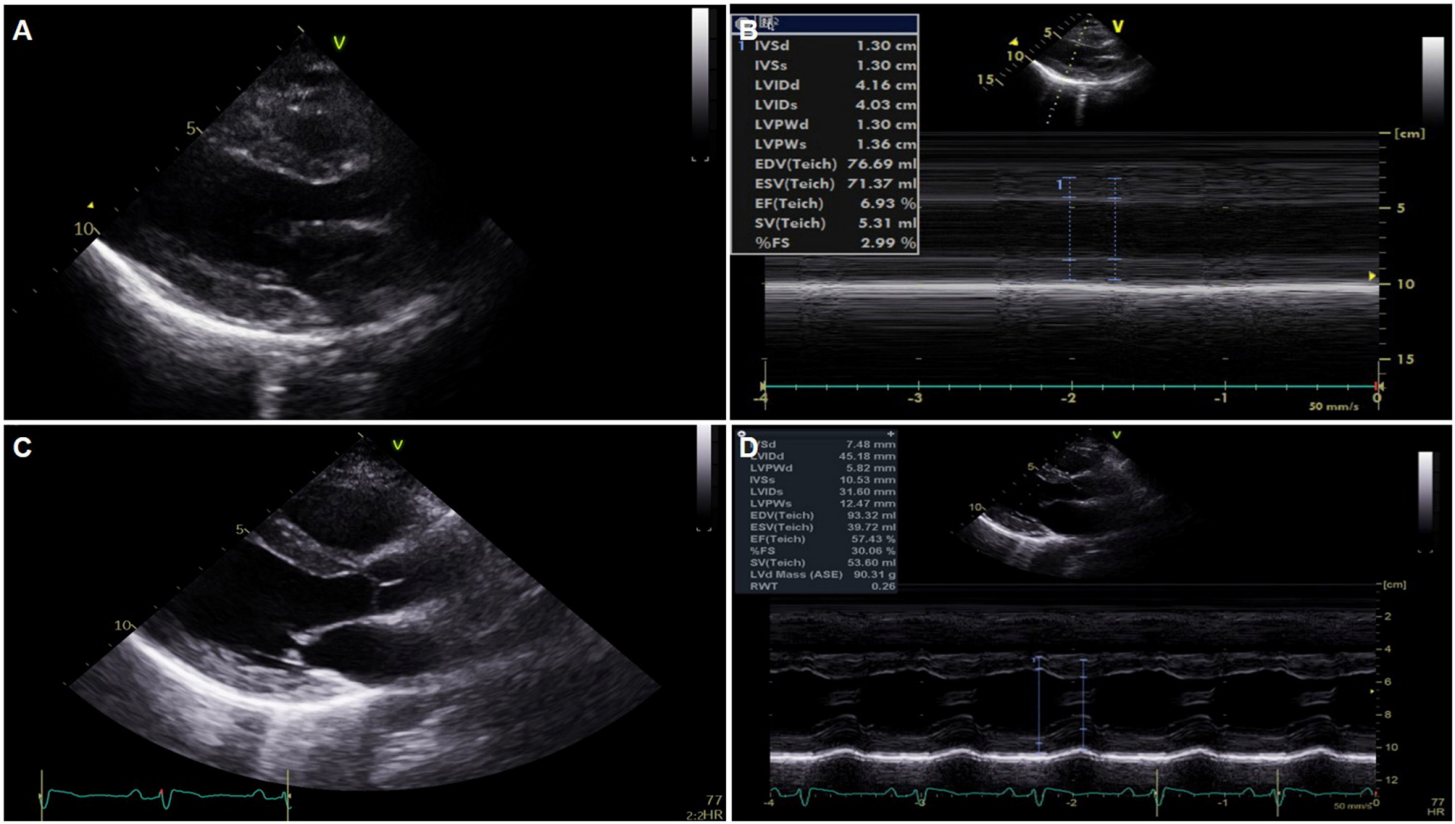
Serial changes of echocardiography. **(A,B)** Global akinesia with severe left ventricular (LV) dysfunction and marked edematous left ventricular wall thickening on portable echocardiography performed just after extracorporeal membrane oxygenation application. **(C,D)** Normalized LV function and wall thickness on pre-discharge echocardiography.

A previously healthy 38-year-old female who received the BNT162b2-mRNA vaccine (Pfizer-BioNTech) 7 days ago presented with ongoing chest pain. The patient did not have any underlying disease, significant family history, and was not on other medications. She was transferred to our heart center because of ST segment elevation on electrocardiogram (ECG), and cardiac arrest occurred immediately after arrival at our center. Despite 10 min of cardiopulmonary resuscitation (CPR), recovery of spontaneous circulation was not achieved. With the aid of extracorporeal membrane oxygenation (ECMO), ECPR (23 French drain cannula into the right common femoral vein and 17 French perfusion cannulas into the left common femoral artery, total CPR time-25 min) was applied. The mental state of the patient was recovered, and there were no remarkable abnormal focal neurologic signs. The ECG showed an extensive ST segment elevation with bizarrely wide QRS complexes in the entire precordial and limb leads except for lead aVR (Figure 1A). It eventually progressed into a total ventricular and atrial electrical standstill lasting for more than 2 h (Figure 1B) and returned to the previous ECG pattern thereafter. Portable echocardiography revealed global akinesia with severe left and right ventricular dysfunction (ejection fraction <10% by visual estimation) and marked edematous left ventricular (LV) wall thickening (13 mm) (Figures 2A,B) (Supplementary Videos 1, 2). Additional cannulation through the left common femoral vein into the interatrial septum *via* atrial septostomy for left heart unloading, coronary angiography, and endomyocardial biopsy (EMB) were done in the catheterization room. There were no stenoses in both the coronary arteries. Laboratory findings showed marked elevation of cardiac troponin I (68.507 ng/ml), N-terminal probrain type natriuretic peptide (32,947 pg/ml), and D-dimer (16.32 mg/L FEU). Fibrinogen assay was normal (205.3 mg/dl). A screening test of severe acute respiratory syndrome coronavirus 2 (SARS-CoV-2) RNA with a polymerase chain reaction was negative, and other viral infection panels and the antibody test to autoimmune disease were non-specific.

On the second hospital day, the follow-up ECG showed a similar extensive ST segment elevation with bizarrely wide QRS complexes, and echocardiography revealed no significant interval change in the previously noted global akinesia with severe biventricular dysfunction.

**Figure 3:**
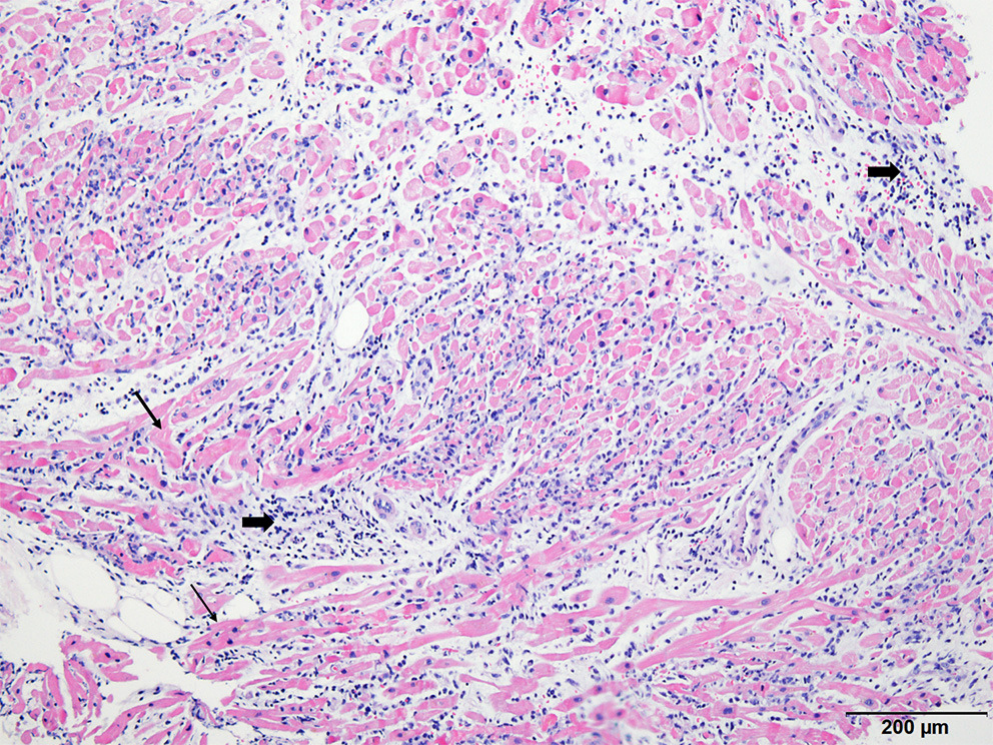
Histopathologic examination of endomyocardial biopsy revealed marked and diffused infiltration of lymphocytes (thick arrow) within the myocardium (thin arrow) (×40, Hematoxylin-eosin stain).

On the third hospital day, echocardiography showed more improvement in both the ventricular function (ejection fraction: 30%) and the decreasing thickness of the LV wall (10 mm). In addition, the ECG showed a more decreased ST segment elevation with the narrowing of QRS complexes (Figure 1C). The patient was extubated on the same day, and ECMO weaning was done on the sixth day. EMB demonstrated compatible findings of acute lymphocytic myocarditis (Figure 3).

**Figure 4:**
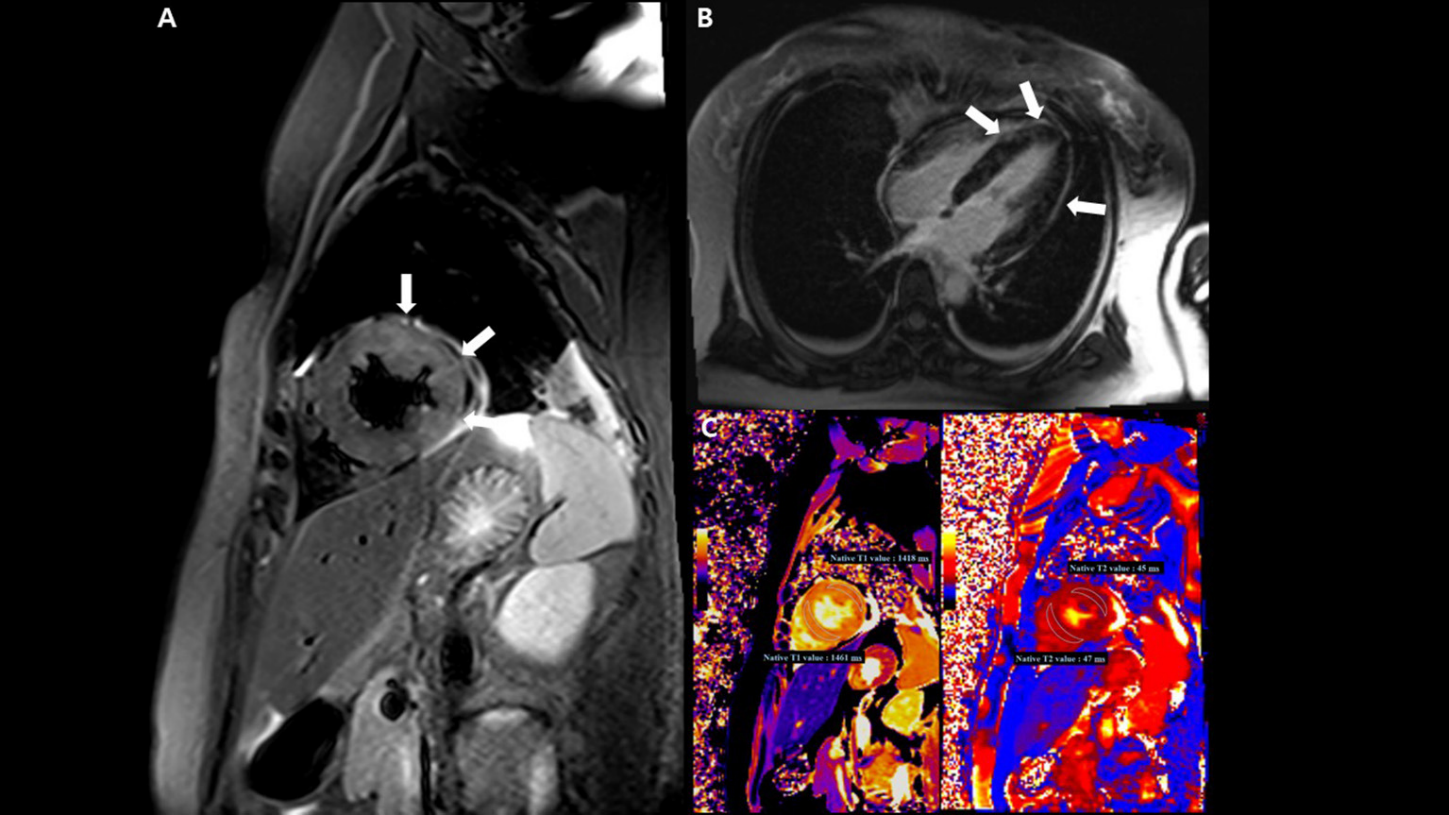
Cardiac magnetic resonance imaging (MRI) findings. **(A)** T2-weighted short TI inversion recovery MRI at 3T in a short-axis view shows multifocal high signal intensities at the mid anterior and lateral wall (arrow) indicating myocardial edema. **(B)** Late gadolinium enhancement imaging in a four-chamber view displays multifocal mid wall enhancement (arrow) indicating inflammatory myocardial necrosis. **(C)** T1 mapping and T2 mapping in a short-axis view show elevated T1 and T2 relaxation times at the mid ventricular level, indicating an acute myocardial injury (regional T1 relaxation time-1,418 ms at the anterolateral wall and 1,461 ms at the septal wall, regional T2 relaxation time-45 ms at the anterolateral wall and 47 ms at the septal wall; institution-specific cutoff values for acute myocarditis- T1 global: ≥1,230 ms and T2 global: ≥36 ms).

On the tenth day, cardiac magnetic resonance imaging was performed, which showed multifocal lesions with high signal intensity in T2 weighted images (Figure 4A) and a positive late gadolinium enhancement (Figure 4B) which implies myocardial edema and necrosis, respectively. In addition, both T1 and T2 relaxation times at the LV wall were also increased (Figure 4C).

Predischarge ECG revealed the normalization of previously noted ST segment elevation and QRS width, and an increased but low voltage QRS complex that remained (Figure 1D). LV function (ejection fraction: 58%) and wall thickness were normalized on the predischarge echocardiography (Figures 2C,D) (Supplementary Videos 3, 4), and the patient was discharged and followed up at an outpatient clinic without clinical events.

## Discussion

The present case gives important clinical messages in the management of the COVID-19 vaccine related myocarditis. First, myocarditis or pericarditis may be a significant differential diagnosis of chest pain after the COVID-19 vaccination. Second, COVID-19 vaccination related myocarditis is not always associated with a mild form of myocarditis or benign clinical course. As demonstrated in the present case, the possibility of disastrous fulminant myocarditis should be carefully monitored. Third, the prompt initiation of a temporary mechanical circulatory support such as venoarterial extracorporeal membrane oxygenation (VA-ECMO) is essential in most critically ill patients with fulminant myocarditis after COVID-19 vaccination, if indicated. Therefore, patients with COVID-19 vaccination-related myocarditis, which is not in the mild form, should be transferred and treated in centers for managing advanced heart failure.

Recently, a CDC Advisory Committee on Immunization Practice suggested a possible association between myocarditis and mRNA COVID-19 vaccines (Pfizer–BioNTech and Moderna) (1). There have been substantial cases of myocarditis or pericarditis after mRNA COVID-19 vaccination reported in the Vaccine Adverse Event Reporting System (VERAS) of the United States (6). According to these reports (1), Bozkurt et al. (7) and Diaz et al. (8) confirmed that myocarditis or pericarditis cases have occurred mostly in male adolescents and young adults, and shortly after (2–3.5 median days) COVID-19 vaccination, and more often after getting the second dose than after the first dose of mRNA COVID-19 vaccination. Most cases showed benign clinical course and responded well to conservative treatment (9–12), and thus CDC continues to recommend mRNA COVID-19 vaccination for individuals 12 years of age and older, given the risk of COVID-19 illness and severe related complications (1). As mentioned above, post-COVID-19 myocarditis has been regarded as a relatively benign one. In many reports, the patients with myocarditis that occurred after vaccination using mRNA COVID-19 vaccines were treated conservatively with or without medication and responded well. In one report, only 10% of patients with post-COVID-19 vaccination myocarditis were treated in an intensive care unit, and all the patients recovered (8). Contrary to most previous reports, the present case demonstrated that the clinical course of the COVID-19 vaccination-related myocarditis is not always benign as reported by others (13, 14), and, currently, only a few case reports of fulminant myocarditis related to mRNA COVID-19 vaccination are available. Without the aid of VA-ECMO, the clinical course of the present case would be fatal. VA-ECMO seems to be more effective to reduce the mortality rate of COVID-19 vaccination related fulminant myocarditis as compared to other cases of fulminant myocarditis (15, 16).

In the present case, a cardiac standstill, which means no electrical activity in both atrium and ventricles, was developed, and it lasted for more than 2 h (Figure 1B). Cardiac standstill, especially a ventricular standstill, is very rare and only a few cases have been described in the literature. It can be presented with either atrial standstill solely (17, 18) or both ventricular and atrial standstill (19, 20). This phenomenon might reflect an extensive inflammatory process that involves both ventricles and the electrical conduction system, and it implies a fulminant feature of myocarditis. In our case, the hemodynamic status of the patient was well-maintained because of ECMO support even though the cardiac standstill persisted for more than 2 h.

In the present case, the diagnosis of myocarditis was confirmed by histology. EMB is a golden standard for the diagnosis of fulminant myocarditis and is highly recommended by expert consensus for clinical purposes (21, 22). It can be seen in cases, reports of post-COVID-19 vaccination myocarditis in the literature that endomyocardial biopsy has been performed in a few cases (13, 14, 23, 24). This lower rate of EMB might be either due to the benign nature of the post-COVID-19 vaccination myocarditis itself, or regional preference of clinical practice for myocarditis. In the COVID-19 era where there are continuous requirements for vaccination for the infection, confirmation of diagnosis by EMB should be more essential for both clinical and research purposes, regardless of reasons for the lower EMB rate. The mechanism of myocarditis following mRNA COVID-19 vaccination, indeed, has not been elucidated and it could be attributable to its relatively rare incidence and benign course. All the factors could contribute toward the lower rate of EMB or autopsy, which produces samples for research.

Although any recommendation for studies to find the etiology of myocarditis has not been cleared so far (21), a thorough search to find any related factors might be more important when diagnosing post-COVID-19 myocarditis for the same purposes. In this case, thorough etiologic tests for myocarditis including viral and autoimmune markers did not reveal any specific cause for myocarditis. As its mechanism is uncertain and there is no specific diagnostic method for this etiology, the etiologic diagnosis of the mRNA COVID-19 vaccine-related myocarditis would be dependent on the manner of exclusion in a case with a temporal relationship, as is the scenario in the present case.

Clinical profiles of the post-COVID-19 vaccination myocarditis in this patient were different in many aspects from previous reports. It was fulminant and occurred after the first dose of the BNT162b2-mRNA vaccine (Pfizer–BioNTech) in a woman in her late thirties. It is unclear whether these different baseline factors are related to the unusual course of the myocarditis in this patient, or not because of the rarity of fulminant myocarditis related to COVID-19 vaccination. We need to wait for further cases to answer this question.

## Conclusion

We reported a case of acute fulminant myocarditis complicated by cardiogenic shock after mRNA COVID-19 vaccination, and the effectiveness of ECMO support was essential for life saving in this special situation. Physicians should be alert to the possibility that myocarditis can rapidly progress after COVID-19 vaccination, and the prompt application of mechanical circulatory support is mandatory if called for.

## Data Availability Statement

The original contributions presented in the study are included in the article/Supplementary Material, further inquiries can be directed to the corresponding author/s.

## Ethics Statement

Ethical review and approval was not required for the study on human participants in accordance with the local legislation and institutional requirements. The patients/participants provided their written informed consent to participate in this study. Written informed consent was obtained from the individual(s) for the publication of any potentially identifiable images or data included in this article.

## Author Contributions

YL, MK, and KK contributed principally to writing the manuscript. I-SJ and YL drafted the first version. MK and YL revised the first draft. KK selected electrocardiogram, echocardiographic images, and drafted the final version of the manuscript. YDC selected the pathologic image and wrote the caption for it. YSC revised the explanation for ECP. JL selected the cardiac MRI images and drafted an explanation for the images. All authors contributed to the article and approved the submitted version.

## Conflict of Interest

The authors declare that the research was conducted in the absence of any commercial or financial relationships that could be construed as a potential conflict of interest.

## Publisher's Note

All claims expressed in this article are solely those of the authors and do not necessarily represent those of their affiliated organizations, or those of the publisher, the editors and the reviewers. Any product that may be evaluated in this article, or claim that may be made by its manufacturer, is not guaranteed or endorsed by the publisher.

## Supplementary Material

Supplementary Video 1 Portable echocardiography on day 2, parasternal long axis view.

Supplementary Video 2 Portable echocardiography on day 2, apical four chamber view.

Supplementary Video 3 Pre-discharge echocardiography, parasternal long axis view.

Supplementary Video 4 Pre-discharge echocardiography, apical four chamber view.
